# Perceptions of the Educational Community on the Inclusion and Presence of Students with SEN in Mainstream Schools: A Mixed Study

**DOI:** 10.3390/children9060886

**Published:** 2022-06-14

**Authors:** Pilar Arnaiz-Sánchez, Remedios De Haro-Rodríguez, Salvador Alcaraz, Carmen Mª Caballero

**Affiliations:** Department of Didactics and School Organization, Faculty of Education, University of Murcia, 30100 Murcia, Spain; parnaiz@um.es (P.A.-S.); rdeharor@um.es (R.D.H.-R.); sag@um.es (S.A.)

**Keywords:** inclusive education, presence, schooling, educational community, special educational needs (SEN)

## Abstract

Achieving inclusive education is a primary challenge for the educational community. Inclusion refers to equal access to education—to the presence, participation and learning of all students. Offering an inclusive education requires all students to share time and space together in the mainstream classroom, that the educational community manifests a positive attitude towards diversity, and that educational centers plan to welcome diversity in their classrooms. The general objective of this study was to evaluate the inclusion of students with SEN enrolled in SOCs in mainstream schools based on their presence, the attitudes of the educational community and the planning processes developed. This was a descriptive study with a dominant status mixed design (QUAN-Qual). The population investigated in this research included the total number of SOCs of the Autonomous Community of the Region of Murcia (Spain) (*n* = 108). The sample obtained comprised 3.891 people belonging to 88 SOCs from 68 educational centers, which implies a confidence interval of 99% (Z = 2.576) and a margin of error of less than 5%. The data collection instruments used included seven questionnaires, adapted for the purposes of the study, for the quantitative phase, and semi-structured interviews, focus groups and discussion groups for the qualitative phase. The study results indicated that the attitudes of the educational community were the main determinant of inclusion. There is a need to reflect on and undertake actions to eliminate existing barriers to the operation of SOCs, since the involvement of students with SEN in the academic and social life of educational centers, and in mainstream classrooms, is not guaranteed.

## 1. Introduction

Since the end of the last century, achieving equitable and inclusive education for all has been the objective of educational policies promoted by most international organizations [1,2]. In this regard, the UN [3], according to the 2030 Agenda and through its 17 Sustainable Development Goals (SDGs), in its fourth objective, highlighted the need to guarantee inclusive, equitable and quality education, and the promotion of lifelong learning opportunities for all. This objective presents inclusive education as one of the greatest challenges to be overcome through the policies and practices of different countries and territories.

Following Booth [4], inclusive education requires the following: equal access to education, presence, participation, and appreciation of diversity and educational achievement for all students in an inclusive environment. For Arnaiz [5] inclusive education must be understood as a project involving professional participation, both social and civic, that requires processes of change and improvement in schools to provide all students with acceptance, learning and well-being. Therefore, inclusive education is understood to involve a continuous process of change, to find better ways to respond to diversity within the mainstream education system, and, in this way, to ensure educational success for all students, without exception.

The levers and barriers which enable or hinder students’ progress, respectively, are related to those elements which favour or hinder the placement or presence of all students in mainstream educational contexts, which is the focus of this article. The principle of presence refers to the sharing of educational settings and time by all students. In this way, an educational system that is committed to inclusion must have a single schooling modality capable of providing and responding to the needs of all and offering equitable and quality education [6,7,8]. However, many students do not enjoy inclusive environments as they are schooled in special education centres, or in specialized units within mainstream centres, such as specialized open classrooms (SOC). These classrooms are aimed at students with SEN who require extensive and generalized support in most of the subjects of the curriculum. SOC’s make it possible for students, traditionally enrolled in special education centres, to attend mainstream schools. In this way, they offer the possibility of sharing times, settings, learning, feelings and experiences with the rest of the students in the mainstream centre, with the aim of making their teaching–learning process as inclusive as possible. However, different research projects have shown that, although these units are located within mainstream centres, there are still barriers to the attendance and participation of students in reference classrooms (RCs) [9,10,11,12,13], bringing into question whether they genuinely offer inclusive education.

In the Region of Murcia (Spain), the context in which this research was carried out, SOCs have been in operation for more than 25 years. These classrooms emerged in mainstream schools with the aim of educating students with disabilities—those who had very serious and permanent impairments and studied in segregated schools—within normalized school environments. The initial objective of these classrooms was, firstly, to enable students with severe SEN to attend school in centres close to their homes (avoiding the need to travel to the nearest special education centre), and, secondly, to improve their inclusion with other young people from the neighbourhood or community. The growth in the use of this type of specialized classroom has been very significant over the last 22 years, having increased from there being only two SOCs in the academic year 2000 to 139 SOCS in the year 2022, and has become an important feature of educational policy. 

It is of the utmost importance to study this type of classroom, since policy for its use is widespread throughout Spain. There have been insufficient studies to determine whether SOCs favour the inclusion of students with SEN, or whether, on the contrary, they support a parallel form of schooling that results in educational exclusion or marginalization.

The presence of all students in mainstream environments is a fundamental lever for inclusion, so this must be the first condition to consider in educational systems that pursue equity and quality [14]. It has been shown that students with SEN who attend a mainstream classroom together with their peers achieve higher educational results than those who attend segregated classrooms in special schools or units [15,16]. The outcomes of projects that have been implemented in Italy [17] and in Canada, where all students with SEN are educated in mainstream schools in their neighbourhood together with their siblings and friends [18], support this statement. In addition, research has shown that students with SEN contribute through their presence in subjects such as music [19], physical education [20] and art and crafts [21], among others. For these projects, there is evidence that the development of this type of schooling promotes acceptance, recognition of differences, academic success for all students and the promotion of inclusive citizenship [22,23]. Therefore, the presence of all students in mainstream learning environments, regardless of their abilities or individual characteristics, is considered to be an essential requirement for the achievement of fully inclusive, democratic and high-quality schools [24,25,26,27].

Diversity in RCs needs to be accompanied by a positive attitude on the part of education professionals and others in the education community. The attitudes of teachers were found to be predictive of the success of inclusion and of the use of inclusive teaching strategies [28]. In this study, in which the confidence of pre-service teachers to apply their professional skills in inclusive classrooms was analysed, it was found that various factors influenced their preparation. The following factors were highlighted: the teacher training programme that was undertaken, the knowledge of legislation and inclusion policy, the relationship and interaction with people with disabilities, the level of confidence of the teachers themselves, previous experience, and, finally, the training provided to work with students with disabilities. According to the study authors, the positive attitudes of teachers towards inclusion contributed to the development of a more inclusive school system. Buli-Holmberg et al. [29] showed that teachers’ attitudes and beliefs can have consequences for educational practice, hence their importance. Furthermore, Nilsen [30], suggested that the attitude of teachers was a possible factor in both the causes and consequences of inclusion; therefore, he recommended the development of a support system for teachers to encourage collaborative work so that inclusion could become a reality. Other studies have highlighted that the attitude of teachers in mainstream centres, and the approach taken to the planning of teaching, can lead to interaction between all students, markedly influencing the development of friendly relations between students with and without SEN, an essential element in the integrated development of every person [31,32,33,34].

In addition to the attitudes of teaching staff having a strong influence, those of RC classmates also have a clear and direct impact on the degree of inclusion of students with SEN in shared settings at common times [35,36,37,38,39]. Suria et al. [40] suggested that it was possible to identify limiting attitudes and beliefs in students without disabilities towards their classmates with SEN, which represented a substantial barrier in the establishment of relationships between all students, especially for those students with greater learning needs, as is often the case for students enrolled in SOCs. To change negative attitudes towards difference, it is necessary to encourage early interaction between children with and without SEN, with encouragement of the presence of all within the RC being a matter of priority for the acquisition of basic learning that can help to improve coexistence, both social and civic [41,42,43].

Inclusive education also requires collaborative pedagogy and practice [44]. Nikula et al. [45] suggested that an inclusive school requires a wide range of actors with shared responsibilities and the collaborative resolution of problems and needs. Other studies have highlighted the need for the involvement and coordination of professionals and for the community in order to respond to the needs of all students and to provide quality education [46,47,48]. Despite this, some studies evidence a lack of coordination, collaboration and communication between specialist teachers and general teaching staff with respect to the attention paid to diversity [49]. Research has found that the origin of these barriers can arise in training [50] and in the approach of educational management teams. In this respect, the role of management teams and their capacity for leadership are key elements in the promotion of a collaborative culture within educational institutions [51].

Offering an inclusive education requires that all students share settings and time in the RC, that the educational community demonstrates a positive attitude towards diversity, and that educational centres develop planning processes which welcome diversity in their classrooms. The general objective of the present study was the evaluation of the inclusion of students with SEN enrolled in SOCs in mainstream schools. The following specific objectives derived from this aim:

1. To analyse the presence of SOC students in the daily life of educational centres.
2. To describe the attitudes towards the presence of SOC students in mainstream schools.
3. To assess the planning processes developed for the presence of SOC students in the RC.

## 2. Materials and Methods

### 2.1. Research Design

This study adopted a descriptive approach. It employed a dominant status (QUAN-qual) mixed sequential explanatory design. A descriptive approach was used to accurately represent the presence of SOC students from different perspectives [52]. A mixed sequential explanatory approach was employed as, in the first phase, quantitative data was collected [53]. To deepen the interpretation and understanding of these data, a second phase was carried out involving the collection and analysis of qualitative data [54].

### 2.2. Participants

The population investigated in this study comprised the total number of specialized open classrooms (SOCs) of the Autonomous Community of the Region of Murcia (Spain) in the 2018/2019 academic year (*n* = 108). These classrooms were located in 82 educational centres in the infant, primary and secondary education stages. Following Hernández et al. [54], non-probabilistic convenience sampling was used. A total of 9375 participants were invited to take part in the study. The final sample comprised 3891 people belonging to 88 SOCs from 68 educational centres, which implies a confidence interval of 99% (Z = 2.576) and a margin of error of less than 5% [52]. The average occupancy ratio of the SOCs was 6–8 students per classroom which were located in a specific classroom within the mainstream centre.

As Table 1 shows, the participants in the quantitative phase comprised 3782 individuals from eight categories of education agent involved in the operation of the SOCs.

SOC students have diagnoses of severe intellectual disability, autism spectrum disorder associated with another type of disability (e.g., intellectual disability, severe communication disorder, severe behavioural disorder), and students with multiple disabilities (i.e., two or more disabilities).

The participants in the qualitative phase were 109 key informants including the following: four professionals from associations and foundations involved in the educational process of SOC students; four professionals from the educational administration involved in the management of SOCs; nine members of the management teams of public and private centres with SOCs for infant, primary or secondary education; 19 SOC tutors; 16 SOC students; and, finally, 57 classmates from the RCs.

### 2.3. Instruments and Techniques of Information Collection

Quantitative phase. Seven questionnaires were designed based on adaptation, for the purposes of the study, of pre-existing questionnaires (i.e., EVABIMUR: questionnaires for the evaluation of specialized open classrooms). These were administered to the management teams of the centres that had SOCs, counsellors, the teaching and specialist team of the SOC, the reference classroom tutor, the tutor of the SOC, families, reference classroom students and SOC students. The objective of the questionnaires was to ascertain the perspective of these educational agents on the functioning and educational provision offered by the SOC in terms of the facilitation of inclusion of students with SEN requiring extensive and generalized support in all areas of the curriculum.

To obtain evidence of content validity for the questionnaires, an expert judgment technique was used, involving the participation of nine specialists in methodology and in the subject matter of the study. These experts completed a purpose-designed content validation template, the results of which made it possible to improve the design of the questionnaires. To assess reliability, Cronbach’s alpha was determined with the following values obtained: management team (0.700), SOC tutors (0.780), reference classroom tutors (0.778), teaching and specialist team (0.818), counsellors (0.727), families (0.950), reference classroom students (0.760) and SOC students (0.801).

As can be seen in Table 2, the questionnaire items were organized along three dimensions: presence, participation and success.

For the purpose of this study, the focus was on the first dimension (presence). This dimension was organized into three categories that are associated with the three specific objectives of this article: (a) presence of SOC students in the mainstream classroom (six items); (b) participants’ attitudes towards the presence of SOC students in the mainstream classroom (eight items); and (c) curriculum planning for the presence of SOC students in the mainstream classroom (eleven items). The questionnaire items comprised closed questions rated using Likert-type scales, with some items rated on a scale of 1–5, some on a sale of 1–4, and some items rated according to a dichotomous scale of 1–2. To explore explanations offered by the participants in greater depth, open questions were also incorporated.

Qualitative phase. The data collection techniques that were used to collect qualitative information from the professional staff and the students were: (a) semi-structured interviews with educational professionals in which issues related to the contributions which SOC students made to the educational centres and the evaluation of these professionals as to which schooling modality was the most appropriate for students with serious and permanent SEN were explored; (b) SOC tutors’ focus groups from which information was collected on the professionals’ evaluation of the presence of SOC students in mainstream contexts and the tutors’ perceptions of the curricular planning used to support the students in the SOC; (c) student discussion groups of the mainstream reference classroom, from which data was collected on the attitudes of the classmates towards the presence in the RC of SOC students; and, (d) the qualitative technique “El Mural de las Situaciones (Situation Mural)” with the SOC students [55] through which information was collected regarding the assessment of the students themselves in relation to their own presence in the RC and the attitudes of the classmates and teaching staff of the reference classrooms.

### 2.4. Procedure

The study was carried out during the 2018–2019 academic year. Prior to this, a study had been carried out focusing on the state of the educational system of the Autonomous Community of the Region of Murcia (CARM) which demonstrated the need to study the schooling modalities of students with SEN, especially in light of the significant acceleration of schooling policies for SOCs by counselling services in the last decade [56]. A project was designed to evaluate the functioning of SOCs as a measure of educational inclusion; a proposal was presented to those responsible for the educational administration of the CARM. After obtaining consent and endorsement to access the educational communities of the SOCs, the project was presented to the participating educational centres. The questionnaires were distributed from the digital platform of the CARM administration with an invitation to participate with informed consent. The research team accessed the completed questionnaires and recorded the information in a statistical programme in order to commence the analysis and to utilise the results. After this analysis, the qualitative phase of field work began with the selected sample. Finally, the process of retrieving the results from the centres and education administration was carried out and the final report was written.

### 2.5. Data Analysis

First, analysis of the data obtained in the quantitative phase was undertaken. This analysis served as the basis for the development of the qualitative phase and subsequent analysis of the information collected during it.

Quantitative phase. The analysis of the quantitative data included, firstly, presentation of descriptive statistics, based on calculation of the frequencies for each of the items comprising the questionnaire scales. Secondly, a study of the data distribution was carried out. It was found that the data did not comply with the assumption of normality (Kolmogorov–Smirnov test, *p* < 0.001) or homoscedasticity (Levene test, *p* < 0.005). Finally, whether there were statistically significant associations between the predictor variables (i.e., gender, age, work experience, type of educational centre, educational stage of the SOC and professional group) and the criterion variables (i.e., the questionnaire items, 4-point Likert-type scale) was tested. An inferential study was performed using non-parametric chi squared (X^2^) tests to study the independence of variables, and the Mann–Whitney U test to compare means, with statistical significance set at α = 0.05. The analysis for this phase was performed using the statistical program SPSS, version 28.

Qualitative phase. The responses to the open questions of the questionnaires were recorded in a data matrix and inductively coded into categories using the Excel program. For the data collected through the qualitative techniques, content analysis was carried out via a deductive process using the Atlas.Ti (V.8) programme. Following a deductive method, a qualitative code-book was created as a reference to guide the coding process, classifying the quotes in relation to the different topics of this research (i.e., reception of students schooled in SOCS, attitudes of the school community towards their inclusion, and planning processes developed). Thus, the qualitative categories and codes used arose from the dimensions defined by the questionnaires used in the quantitative phase, since the purpose of the qualitative phase was to explain, triangulate and relate the different sources of information.

## 3. Results

### 3.1. Objective 1. To Analyse the Presence of the Students of the Specialized Open Classrooms in the Life of the Centres

The presence of SOC students in mainstream classrooms and in the life of the educational institution is the first issue to consider when considering inclusion. In this regard, the management teams indicated that the presence of SOC students in mainstream classrooms occurred to varying degrees. It was reported that a total of 49.5% of SOC students shared settings and timetables with their peers in the mainstream classroom more than four times a week, 31.4% three to four times, and the remaining 19%, only one or two times. The participation of these students in complementary activities was also reported to be diverse. The centre participation by SOC students was reported to be as follows: more than four times a week in these activities (48.6%), three to four times (18.7%), one to two times (29.9%), and, in a small number of cases, never (2.8%).

The percentage presence of SOC students in mainstream classrooms was reported to be: physical education (62.9%), music (43.5%), religion or alternative subject (39.8%), and art and craft (31.5%). The tutors of the mainstream classrooms indicated that they also dealt with other subjects not explicitly referred to in the guidelines, with the percentage presence of SOC students as follows: Spanish language and literature (11.1%), mathematics (10.2%), social sciences (8.3%), natural sciences (8.3%) and English (8.3%).

As shown in Table 3, these subjects were taught in different locations, the mainstream classroom being the most common.

With respect to SOC students being together with their peers at break times, the majority of RC students (45.2%) stated that they were not with their SOC peers, followed by those who stated that SOC peers were sometimes alone (41.6%), with a minority of students claiming to mix with their SOC classmates (13.2%).

In the second phase of the study, the participating professionals corroborated that the presence of the SOC students in common locations and times with the rest of the RC classmates was not always guaranteed. The comments expressed supported the results obtained:

*On many occasions, the SOC children remain isolated in the specialized open classroom and without interaction with the rest of their schoolmates. That is why I say that the specialized open classroom usually becomes a closed classroom. (Professional, president of an association for people with disabilities).*

*A student with a serious disorder and aggressive behaviour cannot be included in the mainstream classroom, in this type of case it is not possible for them to be present with the rest of their classmates. (Professional, Educational Administration).*

In addition, this group commented on the lack of human resources as one of the main causes of the low presence of SOC students in mainstream classrooms:

*The first thing we find, at a quantitative level, is that the level of support for SOC students in the RC is scarce. We only have Therapeutic Pedagogy teachers (PT)* *—specialists in attention to diversity* *—on a part-time basis. In addition, we have one PT for a very high ratio of students with SEN. Thus, children are cared for in groups and do not receive individualized care. (Professional, president of an association for people with disabilities).*

*Attention to all the students within the mainstream classroom is very difficult. We cannot forget that the teachers’ time is limited and that time must be distributed among all the students of the centre. (Professional, headteacher of secondary school with SOC).*

The professionals also indicated limitations in the availability of material resources in the reference classrooms:

*The teaching staff who are tutors of the RCs feel there is a lack of material resources to attend to diversity, which makes it difficult to respond to the students of the SOC. (Professional, headteacher of a primary school with SOC).*

The students of the SOC asked for more time in the RC, as did their peers in the reference classrooms:

*I would like the SOC classmates to come to the reference class for longer periods. I think they spend very few hours with us. (Student, secondary reference classroom).*

*I think that the SOC classmates should spend more time with us in the reference classroom, because I think that is what is best for their learning and for us all to improve coexistence. (Student, secondary reference classroom).*

**Figure 1:**
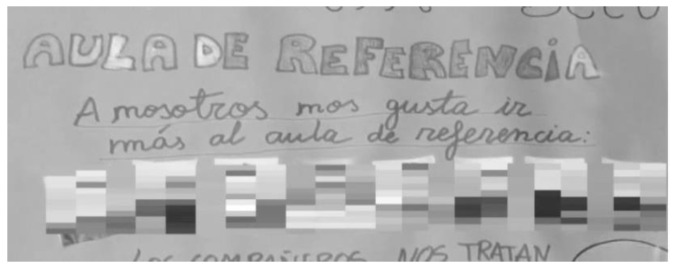
In this quotation graphic, the students of a secondary SOC affirm that they would like to attend the reference classroom more often alongside the rest of their classmates.

*Quotation graphic* (see Figure 1) *(Student, specialized open classroom, secondary):*

### 3.2. Objective 2. This Describes the Attitudes towards the Presence of SOC Students in Mainstream Schools

Most of the professionals expressed a positive attitude towards the students of the SOC, since they considered that this enriched the life of the centre (94.0%). There were significant differences in the assessment made by the different professional groups (χ^2^ (4) = 16.259; *p* < 0.004). Differences were observed between the reference classroom tutors and the SOC tutors (*p* < 0.006), and between the counsellors and the SOC tutors (*p* < 0.032). All the professional agents perceived the SOC to be a valuable measure, with the SOC tutors having the most positive assessment (X = 3.75), followed by the counsellors (X = 3.50) and the reference classroom tutors (X = 3.43).

In addition, statistically significant differences were also observed depending on the type of educational centre (χ^2^ (1) = 46,447.500; *p* < 0.001). An analysis of these differences showed that, in the specialized open classrooms of the state-assisted and private centres, the mean was higher (X = 3.83) compared to that of the state educational centres (X = 3.46). Thus, the state-assisted and private centres displayed more positive attitudes towards the benefits of this specific measure.

Despite the above, the qualitative information that was collected on attitudes of the teaching staff towards the presence of the SOC students in the RCs, indicated some negative attitudes:

“The main flaw is that the teachers of the reference classrooms do not have the necessary commitment to be able to work with the children of the SOC. (Professional, headteacher of primary school with SOC).

There is a clear lack of commitment on the part of some RA teachers. The teachers of the reference classrooms usually defend themselves with the fact that they are not professional specialists in attention to diversity and, therefore, they assert that they do not have to work with the students of the SOC. (Professional, tutor of SOC, primary).

Schools can give as much as they can and no more. Although the regulations tell us that there must be inclusion in mainstream classrooms, sometimes it is possible, but at other times it is impossible for such inclusion to exist in the RA. (Professional, headteacher of a primary school with SOC).”

The families expressed very positive attitudes (90.6%) towards the SOC in the mainstream centres. More than 50% of families expressed feelings of rejection towards their children being schooled in special education centres, with no significant differences with respect to educational stage or the type of educational centre (Table 4).

The attitudes of the students in the reference classrooms towards the presence of the SOC classmates in the RC was very positive. A total of 76.1%of RC students affirmed that they liked their SOC colleagues attending the RC (Table 5).

Regarding the attitudes of the SOC students themselves, the majority (91.2%) indicated that they liked going to the RC, while the percentage that indicated the opposite was minimal (8.8%). In addition, 87.7% of the students affirmed that they liked being with their RC classmates and 93.0% indicated that they liked the teachers in the classroom (Table 6):

In line with the above, the attitudes of the students towards the presence of SOC students were very positive:

“I like that my colleagues from the SOC come to the classes because I learn what these people are like and how I should treat the diversity of people who are in the street, outside the school. Therefore, I learn a lot from each of them, how to talk to them, how to treat them, etc. Then, if I meet another person like them in the street, then I know how to act correctly. (Student, secondary reference classroom).

I do want the SOC child to be here in the class playing and working with all of us. I like them a lot and they greet me when they see me. In addition, they also like to be with us a lot, they are always happy and smiling when they see us. (Student, primary reference classroom).”

**Figure 2:**
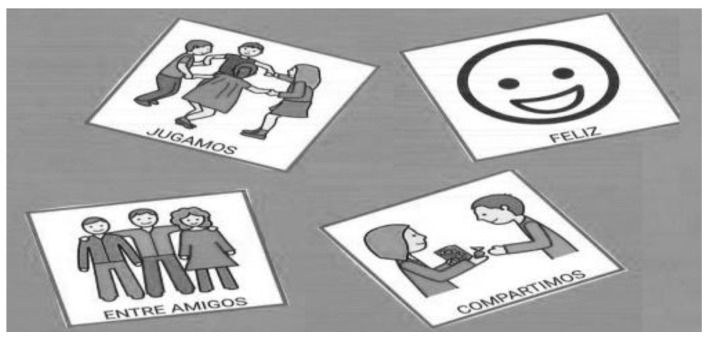
In this quotation graphic, a primary SOC student offers a positive assessment towards his classmates in the reference classroom when present with them. This indicates that they are all friends and that they are happy being together.

Quotation graphic (see Figure 2). (Student, specialized open classroom, primary):

### 3.3. Objective 3. Assess the Planning Processes Developed for the Presence of SOC Students in the RC

In the decision-making process regarding the assignment of SOC students to a reference classroom, the opinions of various teaching and non-teaching agents were considered. In relation to decision making, the SOC tutors (85.0%) and heads of studies (74.8%) were those who participated the most. It is worth highlighting that the counselling departments (48.6%), the reference classroom tutors (34.6%) and the rest of the SOC teaching team (41.1%) participated little. Counselling services (15.9%) and families (23.4%) were the least active.

From the point of view of the management team, the main criteria for assigning SOC students to a reference class were their level of curricular competence (30.5%), as well as the likelihood of social integration of the students within the reference class (24.5%). Assignment to a reference classroom by chronological age was accorded little prominence (19.8%), as were the attitude of the reference classroom tutor (13.1%) and questions of an organizational nature (13.5%).

Most management teams (60.7%) indicated that coordination procedures were established in the diversity attention plan (DAP) with respect to the activities that SOC students carried out in the reference classroom. However, some management teams surveyed stated the opposite (39.3%). These processes were diverse and could be organized into two categories: (1) according to the agents that participated in the coordination; and (2) according to the education component catalysing the coordination procedure.

In relation to the first category, the coordination of activities was carried out by the SOC and RC tutors, in coordination with the head of studies. Other responses indicated that coordination was achieved by the entire SOC teaching team. Less frequently, there were also procedures for coordinating activities through teaching coordination bodies, such as: the Pedagogical Coordination Commission (PCC), the year or stage pedagogical coordinators and the diversity support team. Finally, individual coordination procedures with counselling services and associations were also highlighted. Professionals highlighted the importance of adequate coordination between educational agents and pointed out that, sometimes, this did not occur in the centres:

*Coordination between professionals (SOC and reference classroom teachers) is very important and sometimes does not exist. I think that more meetings should be held between all the professionals who are part of the teaching team of the specialized open classroom (SOC). (Professional, headteacher of a primary school with SOC).*

*The fundamental thing when it comes to working adequately with the children of the SOC is the coordination between all the professionals, but we—the teachers—do not carry out this coordination well. (Professional, primary SOC tutor).*

In relation to the second category, most responses referred to the fact that the coordination procedures that were reflected in the DAP referred to activities in which SOC students could be present with their reference group. However, the responses of the management teams referred to other elements including: the measures of attention to diversity that were to be used in the sessions when the SOC students were in the reference classroom, the situations in which they attended, the hours of attendance of the SOC students in the reference classroom (5.88%), the initial evaluation of students to assess their level of curricular competence (CL) and their SEN, the organization of internal resources and the monitoring, review and evaluation processes.

As can be seen in Table 7, 76.4% of the tutors in the reference classroom stated that they have pre-planned activities for when the student in the specialized open classroom attended the reference classroom. The majority of RC tutors designed activities (72.2%), methodologies (62.3%) and teaching materials (54.6%) adapted to or that facilitated the participation of SOC students in their classes.

This planning was based essentially on the design and adaptation of activities and materials, with some collaboration from the specialized open classroom tutor, the principal task being guidance and advice on specific methodologies. Among the criteria mentioned by the reference classroom tutors for SOC students was the sharing of certain contexts/activities with the rest of the reference group, the development of socialization (94.4%), the promotion of integration (88.0%) and the opportunity for participation (85.2%). By contrast, the least frequent criterion referred to the student’s curricular adaptation (22.2%).

## 4. Discussion and Conclusions

Ensuring lifelong inclusive education for all represents a constant challenge for global educational policies [1,2,46]. The paradigm to follow for educational cultures, policies and practices is that of inclusion [5]. For this reason, this article evaluated the inclusion of SEN students schooled in SOCs in educational centres and in RCs.

The findings of this study showed that students enrolled in SOCs are present to varying degrees in the life of the centres and RCs, as pointed out by Arnaiz & Caballero [14]. The subjects where they share learning processes with their peers are physical education, music, religion or an alternative subject, and art and craft. As in previous studies [19,20,21], the great support provided by these subjects in the process of inclusion was highlighted. The presence of SEN students in other subjects (e.g., Spanish language and literature, mathematics, social sciences, natural sciences and English), which are allocated a greater number of teaching hours per week, occurred in a small percentage of cases. This represents a clear barrier to inclusion that, in turn, perpetuates exclusion and the existence of parallel schooling models in supposedly inclusive environments [10]. We should not forget that presence is an essential requirement for the development of fully inclusive and democratic schools [6,7,8].

As in other studies [9,11,12,13], teachers sought to justify the sparsity of presence of SOC in the RC, in terms of the limited number of hours and curricular subjects, by alluding to the lack of human and material resources needed to facilitate the attention and response to these students in the RC and in the academic and social life of the centres. We should not forget the studies that have shown that children who attended mainstream educational centres with their peers obtained better results than those who were schooled in segregated classrooms [15,16,17,18]. In this sense, the SOC cannot be considered to be an inclusive measure if it does not fully comply with the principles of presence, participation and the learning of these students [4].

Despite this sparse presence, the students of RCs and the SOCs indicated their desire to be present in the same setting, to share timetables and to participate in the same activities with appropriate adjustments. This stance promotes acceptance, recognition of differences, and the promotion and learning of truly inclusive citizenship, as has been shown in different studies [22,23]. Therefore, it is necessary to increase the presence of these students in the life of the centres and classrooms, to eliminate the barriers that prevent inclusive education from becoming a reality [24,25,26,27].

Presence is the first step on the journey towards inclusion, but attitudes towards presence represent one of the fundamental conditions for its implementation. For this reason, the attitudes shown by different groups towards the presence of SOC students in centres and classrooms were discussed, pointing out that these represent a lever for the promotion of inclusive education [29,41,42,43]. General teaching staff, on the whole, showed very positive attitudes towards the presence of SOC students, pointing out the value that they added to the life of the centres. Positive attitudes were observed to a greater extent amongst tutors of SOCs and in the general teaching staff in state assisted/private centres. It should not be forgotten that teachers’ attitudes are predictors of the success of inclusion and the use of inclusive teaching strategies [28], and represent both a possible cause and consequent outcome of inclusion [30]. Hence, the emotional dimension in inclusive schools must also be considered, where the diversity of human beings is valued, and the well-being of every person is respected and sought [40]. The centres studied had positive attitudes and this is essential for the promotion of relationships and friendship between students with or without SEN [31,32,33,34].

Only a small percentage of the teaching staff considered that the SEN students brought little added value to the life of the centres. Examination of qualitative quotes showed that, even today, there are teachers who consider that students with serious disorders and aggressive behaviour cannot be included in the mainstream classroom, but must be in separate special education classrooms, suggesting a narrow view of what inclusion implies. Thus, some teachers considered that inclusion was not possible for all students, but only for those who had fewer difficulties and did not cause disruption in regular classrooms. This may represent a substantial barrier to progress towards inclusive education systems for all. These negative perceptions on the part of the teaching staff towards the inclusion of the students of SOCs could be related to some of the factors highlighted by research [28], among which we suggest a possible lack of confidence of teachers to attend to diversity, and the limited previous experience of teachers with students with disabilities or insufficient training to respond to the learning needs of students with SEN in the mainstream classroom in this Spanish territory. 

The students of the SOC and the RC showed positive attitudes towards sharing settings and time in mainstream environments. The RC students expressed their pleasure at the presence of the SOC students in the RC, and, in turn, SOC students showed their willingness and pleasure in attending it, being with their peers and with the teaching staff. These student attitudes represent a lever for inclusion and directly affect the degree of inclusion of students with SEN in the shared settings and times in the centre, as other studies have shown [35,36,37,38,39].

Finally, in relation to the attitudes shown by the educational community, families are mostly in favour of these classrooms and of this type of schooling, indicating that they are against the schooling of their children in segregated environments. These results support the thesis of various authors and studies, of the need to educate all students in the same centres, and the need to abandon specific schooling modalities that perpetuate the segregation and exclusion of some students, especially those who are most vulnerable [46].

The presence of SOC students in schools and RCs requires not only positive attitudes, but also the development of planning processes [44,45]. This has been one of the objectives addressed in this study. The process of assignment of SOC students to the RCs involves the participation of the majority of the tutors of the SOC in the centres and the heads of study. Other professionals, while being involved in decision-making, showed a low level of participation (e.g., counselling service, RA tutors, teaching teams and families). The criteria that justify the assignment to a specific RC are related to the level of curricular competence of the students and the attitudes of their RC classmates towards facilitating their social integration.

It is worth noting the low participation of all professionals in the planning processes which are essential to guarantee inclusion [46,47,48]. Similarly, it was clear how important the role and functions of management teams [51], teachers, professionals from educational counselling services, as well as professionals from complementary educational care were in these processes. It was striking that coordination and communication processes between SOC tutors and RC tutors were rare, which implies a clear barrier to inclusion. It is necessary to have an organization and a learning plan that ensures a quality educational response for each student, without exception.

In this regard, it is clear that there was a lack of a collaborative culture in responding to the needs of all students in the centres [49], as well as a lack of relevant teacher training to enable them to meet the challenge of making inclusion a reality [50]. The results obtained showed that there are barriers related to insufficient coordination between the professionals of the specialized open classroom and the RC with respect to establishing common pathways of educational action for students with SEN [10]. The results also suggest that there was limited involvement of the RC teachers with the SOC students and a lack of planning and adaptation in the educational curricular response that these students required.

Everything expressed above leads to the conclusions of this study described below in relation to the formulated objectives.

The presence of SOC students in the academic and social life of the centres and RC was not consistent in the study carried out. Students with SEN, who have had the good fortune to attend a centre where diversity is celebrated and inclusive educational practices are developed, are present on a significant number of occasions in the activities of the centres and the RC, in contrast to others where their presence is scarce and relegated to the SOC. The low presence of SOC students in RC activities is justified by professionals based on the scarcity of human and material resources, which represents a clear barrier to inclusion. This reality is perceptible in the different educational stages and regardless of the type of the centre. The voices of all students demand a greater presence in the RC, with SOC students and their peers in the RC expressing a desire to be together, which itself represents an important lever towards inclusion.

Despite having a diverse presence of SEN students, depending on the type of educational centre, the attitudes amongst general teaching staff towards the presence of students with SEN were very positive. The teaching staff believed that the presence of SOC students in the centres enriches them—this evaluation was particularly positive in the case of SOC tutors and was found in both private and state-assisted centres. RA students also expressed the benefits of this presence, considering that it favoured knowledge, acceptance and respect for difference. Families also expressed positive attitudes towards this type of schooling, since they considered that it made it possible for their children not to have to go to school in a specific, segregated and exclusive environment, such as special education centres. For all these reasons, the attitudes present in the educational community towards the SOC constitute a lever towards inclusion.

The planning processes developed for the presence of the SOC students in the centres and RC revealed, in the majority of cases, an absence of the teamwork needed to build an equitable and quality education for all that does not exclude some needs and some students due to their specific characteristics. The assignment of these students to the RA falls, almost always, to the tutor teacher of the SOC and to the head of studies of the centre, with little participation of the educational counselling professional or the tutors of the RC. The main decision-making criteria in the assignment of SOC students in the RC was the level of curricular competence and the possibilities for social integration in the classroom.

With respect to the overall objectives of this study, we have highlighted the levers and barriers to the inclusion of students with SEN enrolled in SOCs in mainstream centres. The study has enabled us to determine that the attitudes present in an educational community are a primary lever towards inclusion. There is also a need to reflect on and carry out actions that can eliminate the barriers present in the operation of SOCs, since the presence of SEN students in the academic and social life of the centres and the RCs is not guaranteed. Many educational institutions lack a collaborative culture that supports coordination and planning procedures to bring about inclusive education. In short, it is worth focusing attention on improving mainstream classrooms so that, little by little, students who are now enrolled in SOCs can attend them, in order to build a fully inclusive education, which will allow the objectives of the 2030 Agenda for sustainable development to be met [3]. 

SOCs cannot be understood as an isolated response to a complex problem, that of exclusion/inclusion. Educational policies are required that are unreservedly committed to inclusion with no nuances and without selection. Educational policy must be oriented towards the provision of the necessary conditions that guarantee an inclusive, equitable and quality education for all students. This requires a transformation of the mainstream classroom into a classroom for all students, far from the dominance of ableism. The provision of resources and the design of policies for the initial and on-going training of teachers—of all teachers, not only specialist support teachers—are inescapable issues. Alongside these aspirations, it is necessary for the management teams of educational centres to apply their leadership skills to achieve inclusion. This leadership should be oriented towards clear commitments and the promotion of processes for educational change. In addition, research must continue adding evidence that can provide guidance on the way forward for the transformation of educational realities towards inclusive learning contexts.

Considering the limitations of our research, we advocate that studies are carried out in other areas within the Spanish context that will allow comparison of the effectiveness of this schooling modality in order to promote the inclusion of students with SEN in mainstream educational environments.

## Figures and Tables

**Table 1:** Sample participating in the questionnaires.

	Sample Invited	Sample Participation				
	Total	Primary	Secondary	State	State-Assisted	Total
Members of Management Teams	164	66 (61.7%)	41 (38.3%)	76 (71.0%)	31 (29.0%)	107
Tutors of specialized open classrooms	108	64 (72.7%)	24 (27.3%)	56 (63.6%)	32 (36.4%)	88
General teaching staff and non-teaching specialists	324	130 (66.3%)	66 (33.7%)	127 (64.8%)	69 (35.2%)	196
Counsellors	82	53 (73.6%)	19 (26.4%)	51 (70.8)	21 (29.2%)	72
Tutors of the reference classrooms	324	78 (72.2%)	30 (27.8%)	87 (80.6%)	21 (19.4%)	108
Family	585	156 (72.6%)	59 (27.4%)	118 (54.9%)	97 (45.1%)	215
Students of the SOC	688	206 (59.4%)	141 (40.6%)	211 (60.8%)	136 (39.2%)	347
Students of the reference classroom	7100	1662 (62.7%)	987 (37.3%)	1438 (54.3%)	1211 (45.7%)	2649
Total	9375	2415 (63.9%)	1367 (36.1%)	2164 (57.2%)	1618 (42.8%)	3782

Source: Prepared by the authors.

**Table 2:** Grouping of the items of the questionnaires by dimensions.

Questionnaire	Items	Dimension 1. Presence	Dimension 2. Participation	Dimension 3. Success
Management team	22	-Presence in mainstream classroom -Attitudes towards presence -Curriculum planning	-Inter-professional coordination -Training of professionals -Assessment on reception -Student voices -Actions that promote participation	-Impact on the academic performance of the rest of the student body. -Benefit for learning for all students. -Acquisition of specific skills in terms of SOC students’ autonomy, communication, socialization, etc.).
Tutor of SOC	24			
Tutor of the reference classrooms	23			
General teaching staff and non-teaching specialists	17			
Counsellor	8			
Family	4			
Student of reference classroom	14			
Student of SOC	31			

**Table 3:** Spaces where the subjects contemplated in the guidelines are taught.

Subject	Teaching Space Mainstream or Reference Classroom	Teaching Space Outside the Mainstream Classroom
Physical Education	60.3%	39.6% Specialized open classroom Gym/sports hall/courtyard Motor skills training room Tennis court
Art	69.1%	30.9% Specialized open classroom Art and craft classroom Music room Workshops
Music	64.3%	35.7% Specialized open classroom Music room
Religion or alternative subject	52.4%	47.6% Specialized open classroom Religion classroom

Source: Prepared by the authors.

**Table 4:** Descriptive statistics in relation to the type of schooling preferred by families.

	Totally Disagree (%)	Disagree (%)	Unsure (%)	Agree (%)	Totally Agree (%)
I consider that the special education center is the best option for schooling my son.	44.5%	13.9%	5.7%	14.8%	21.1%
I consider that the specialized classrooms are the best option to educate my children	1.9%	2.8%	4.7%	26.3%	64.3%
I consider that the mainstream centres are the best option for schooling my child	16.1%	18.5%	13.7%	21.0%	30.7%

Source: Prepared by the authors.

**Table 5:** Descriptive statistics of the attitudes of the students in the reference classrooms.

Item	Percentages		
	No	Sometimes	Yes
I like that the schoolmate from the specialized open classroom comes to my class.	3.3%	20.6%	76.1%

Source: Prepared by the authors.

**Table 6:** Descriptive statistics of the attitudes of the SOC students towards their presence in the RC.

Item	Percentages	
	NO (%)	YES (%)
I like to go to the reference room	8.8%	91.2%
I like to be with the classmates in the RC	12.3%	87.7
I like the teachers of the RC	7.0%	93.0%

Source: Prepared by the authors.

**Table 7:** Planning of activities for SOC students.

Item	Yes (%)	No (%)
There is prior planning of activities when the student from the specialized open classroom attends the reference classroom	76.4	23.6
Design activities adapted to the characteristics and needs of the students in the specialized open classroom	72.2	27.8
Design teaching materials adapted to the needs of students in the specialized open classroom	54.6	45.4
Do they design methodologies adapted to the characteristics and needs of students in the specialized open classroom?	62.3	37.7

Source: Prepared by the authors.

## Data Availability

The data presented in this study are available on request from the corresponding author. The data are not publicly available for privacy reasons.
